# Just In Time! Assessment of Internal Medicine Resident Point of Care Ultrasound (POCUS) Attitudes and Behaviors After Spaced Intervention at Two Residency Programs

**DOI:** 10.24908/pocus.v9i2.17635

**Published:** 2024-11-15

**Authors:** Kevin M Piro, Patricia A Carney, Christopher J Smith

**Affiliations:** 1 Division of Hospital Medicine, Division of General Internal Medicine, Oregon Health & Science University, School of Medicine Portland, OR USA; 2 School of Medicine, Division of Hospital Medicine, Oregon Health & Science University Portland, OR USA; 3 Division of Hospital Medicine, University of Nebraska Medical Center Omaha, NE USA

**Keywords:** Point-of-Care Ultrasound, Internal Medicine, Curriculum Development, Graduate Medical Education, POCUS

## Abstract

Point of care ultrasound (POCUS) is a complex psychomotor skill that requires scaffolded support for skill acquisition. However, the effect of spaced curricular elements on learner POCUS behaviors are not clearly understood. Using a multi-site observational cross-sectional survey study, we measured resident baseline POCUS use, behaviors, and attitudes and then implemented POCUS workflow and just-in-time POCUS curricula during internal medicine resident ward rotations and assessed changes. Self-reported personal and team POCUS use and documentation habits all improved between baseline and the just-in-time teaching. Personal POCUS use correlated with team POCUS use (ρ=0.431; p<0.001) and co-resident POCUS use (ρ=0.242; p=0.035). Attending POCUS use correlated with team POCUS use (ρ=0.523; p< 0.001), but not personal use. Overall, we found moderate, but statistically significant, improvements in reported resident and team performance of POCUS and documentation habits, suggesting that just-in-time interventions may promote POCUS use. Co-learning also appears to be a key influencer for POCUS use.

## Introduction

Point of care ultrasound (POCUS) holds exciting potential to improve bedside care by disrupting traditional diagnostic pathways 1, 2, 3, 4, 5, 6. It could improve providers’ diagnostic and prognostic capabilities as well as the therapeutic relationship by fostering physicians’ return to bedside care. However, the merits of POCUS do not come without associated growing pains. As professions seek to encourage the adoption of POCUS skills, the pace at which this is occurring has caused some alarm in the medical community. This has led for calls to provide appropriate safeguards for skills development, like ensuring that requisite training is provided and systems to support documentation and asynchronous feedback are in place 7, 8. What this training actually consists of remains hotly debated with specialty groups calling for investigation into the pedagogical methods used to teach POCUS 9, 10.

Within internal medicine (IM), trainees have expressed sustained interest in POCUS, leading to rapid growth in curricula 11, 12. Workshop experiences have become widely popular, due to ease of logistical management. However, growing evidence suggests that these programs are insufficient to fully establish competent POCUS-informed care 13, 14, 15, 16, 17. As IM trainees move beyond workshop experiences into clinical environments, gaps exist in current knowledge related to resident POCUS practice patterns (e.g., frequency of use, type of use), attitudes (e.g., confidence in skills, perceptions of clinical utility, influencers of POCUS use), and behaviors (e.g., the ability to seek out additional confirmatory testing or supervision, when indicated) – especially as POCUS devices become smaller, more accessible, and more affordable 5. However, access alone can be insufficient to improve POCUS use and image quality 18, 19. 

Spaced Repetition Theory posits that “forgetting occurs exponentially” and “retention of information needs to be an active process,” providing a framework for conceptualizing both learner POCUS skill acquisition and loss 20. Adding spaced components to a curriculum has shown marked improvements in both knowledge and psychomotor skill retention 21, 22. What is not known, however, is how residents’ POCUS use is affected by this training model when it is delivered during routine clinical care. 

In this study, we explored the impact of a just-in-time spaced POCUS curriculum on IM residents’ POCUS behaviors and attitudes using assessments based on Kirkpatrick’s Model of Learning Outcomes 23. First, we established baseline practice patterns for POCUS use and documentation behavior; then we provided POCUS workflow training activities; and finally, we provided a Just-in-Time (JIT) POCUS curriculum to residents. We assessed both resident learning (Kirkpatrick Level 2 – Learning), and self-reported practice patterns and documentation habits (Kirkpatrick Level 3 – Behavior), while simultaneously assessing influencing factors.

## Materials and Methods

### Setting, Participants & Study Phases

We used an observational cross-sectional study design at two institutions, Oregon Health & Science University (OHSU) and the University of Nebraska Medical Center (UNMC), to assess residents on hospital ward rotations. Characteristics of these programs and their POCUS curricula are presented in Table 1. Participating residents included all categorical and off-service residents on IM inpatient ward rotations. Resident teams included a senior resident and one or more interns under the supervision of a faculty physician. The inpatient rotation structure differed slightly between programs. 

This study was conducted between September 2020 and June 2021 and involved three phases. Phase 1 (Observation, Obs) assessed baseline POCUS attitudes and behaviors (September-December). Phase 2 (Workflow, WF) involved assessment of an implemented workflow curriculum where residents received passive instruction on proper methods to archive acquired POCUS images and document their findings (January-February). Phase 3 (Just-in-Time, JIT) assessed a just-in-time curriculum with improved access to handheld POCUS devices (March-June). 

**Table 1:** Site Characteristics of Internal Medicine Residency Programs Participating in Study

**Characteristics **	**OHSU**	**UNMC**
Total Number of Categorical IM Residents † PGY1* PGY2 PGY3	(n=109) 36 36 36	(n=90) 30 30 30
Practice Setting	Urban Academic	Urban Academic
Affiliated VA Health Center	Yes	Yes
Inpatient Rotation Structure		
Number of teams	5	5
Duration	3 Weeks*	1 Month
Team Members	1 Senior Resident, 1 Intern	1 Senior Resident, 2 Interns
Off service residents	Yes Anesthesiology	Yes Psychiatry& Anesthesiology
Residency POCUS Program		
Fundamental “Boot Camp”	Yes-Fundamentals, Cardiac, and Lung	Yes– Fundamentals, Cardiac, and Lung
Additional POCUS Workshops	No	Yes –POCUS-guided procedures and abdominal POCUS
POCUS Elective	1-3 Weeks	1 Week
POCUS Fellowship	Yes	No
FTE for POCUS Program Director	0.2	0.15

* Some residents are off-cycle and may complete a shortened rotation of 1 or 2 weeks.

†Residents in preliminary internship training were excluded from analyses because they were only included in PGY-1 and have different motivations for POCUS training.

Abbreviations: OHSU-Oregon Health & Sciences University, UNMC-University of Nebraska Medical Center, POCUS-point of care ultrasound, VA-Veteran Affairs, FTE-Full-time equivalents

### Baseline POCUS Curricula

Both OHSU and UNMC offered a workshop experience for categorical first year residents, similar to previously described curricula (Table 1) 13, 24. At OHSU, all residents except for non-categorical residents (no more than one per month) and at UNMC, all IM and Medicine-Pediatric residents participated in workshop curricula. At OHSU, interns participated in an introductory workshop during “Intern Intensive Week” in August, which included 2–4-hour sessions covering ultrasound fundamentals, cardiac, and lung exams. Over the course of the year, interns also received POCUS-procedural training during their 1-week procedure rotation. At UNMC, interns participated in three workshops during the year. The POCUS “boot camp” occurred as part of their residency on-boarding in late June and covered ultrasound fundamentals, cardiac, and lung exams. They also participated in a POCUS-guided procedural workshop in the fall and anabdominal exam (kidney, bladder, aorta) workshop in the spring. Both programs similarly offered a POCUS elective for residents. 

### Intervention Activities

Workflow Curriculum: POCUS Training Directors created a standardized workflow training program that instructed residents in institutional standards for POCUS image archival and documentation. Both institutions utilized Qpath E software (Telexy Healthcare Inc., Maple Ridge, British Columbia) for image archival. The process for POCUS exam documentation at OHSU required use of a SmartBlock macro element embedded within standard clinical documentation within the electronic medical record (EPIC Systems Corporation, February 2020 version, Verona, WI). Workflow training was delivered to residents via oral presentation with slides during ward orientation and an email with a short instructional video, and with twice weekly reminders during didactic sessions. 

At UNMC**, **POCUSdocumentation took place within Qpath E. Residents received training on this workflow during the required monthly rotation orientation. An instructional video and step-by-step instructions were also posted to the residency’s online learning platform and in resident team rooms.

At the time of the study, there was no workflow in place for team attendings (the medicine service supervising physician) to review ultrasound images.

### Just- in-Time Intervention

Starting in March 2021, residents on wards services received one structured JIT hands-on POCUS didactic session (Figure S1) that provided instruction on lung, inferior vena cava, and internal jugular vein ultrasound. At OHSU, this one-hour session was led by faculty and was performed in-situ on patients under the resident team’s care. The JIT sessions were completed within the first week of the rotation and had a ratio of two learners to one facilitator. These occurred at roughly three-week intervals based on the rotation structure. At UNMC, the JIT training took place during two required one-hour educational conferences held on subsequent days within the first week of the rotation**. **Participants practiced POCUS exams on one another with a ratio of 3-4 learners per facilitator. This training was delivered at a monthly interval. While the majority of residents attended the JIT, formal attendance was not tracked for reporting. At both sites, facilitators were POCUS-proficient and included two of the study authors (KP, CS).

During this phase, two handheld units were placed into the resident team rooms for use at both institutions.At the conclusion of the rotation, residents at both sites received a survey with assessment variables based on training objectives as described below.

### Instrument Design, Pilot Testing & Administration

POCUS study site investigators (authors KP and CS) created a 25-item survey (Figure S2**)** using an iterative process to investigate residents’ demographics, previous POCUS exposure, POCUS access, personal and team use patterns, and modifying factors. This process was informed by a previous survey 11. The survey was pilot tested for clarity, usability, and face validity by residents and POCUS experts at OHSU. Feedback obtained in the pilot test led to improvements and re-testing. The same survey was administered during each phase.

At OHSU, the survey was administered in-person 2-3 days prior to the conclusion of rotation. If a resident was unavailable to receive the survey in-person, a link with access to the survey online (Qualtrics) was sent via email. At UNMC, an electronic survey was created in Microsoft Forms. Residents were sent an email link during the final week of the rotation with a reminder email several days later. The survey asked residents to reflect upon their wards experience when responding.

At OHSU, all study activities were approved by the Institutional Review Board (OHSU IRB #16922). At UNMC, the study was reviewed by their Institutional Review Board and determined to be a quality improvement project.

### Data Analyses

Descriptive statistics, including frequencies, means, medians, standard deviations and interquartile ranges were calculated to assess the shape of the data according to each study phase. Means and medians were similar enough that we reported means and standard deviations (SD). We assessed whether differences existed in survey findings across institutions, and because findings were not statistically different, we pooled the data and assessed study findings across the three study phases. We created a summary comfort score by summing respondent scores from the five comfort variables that could range from 5-25. 

Chi-Square was used to assess differences for categorical variables across study phases. The non-parametric Kruskal-Wallis test was used to assess continuous data across the three study phases. Spearman’s correlation coefficient was used to assess correlations. We reverse scored the Perception of POCUS Usefulness scale so scale directions were consistent. A value between 0.50 and 1.00 indicates a strong positive correlation, 0.30-0.49 medium correlation and below less than 0.29 is considered a small correlation. Alpha was set at 0.05 for determining statistical differences. All tests were two-tailed.

## Results

Overall, 110 survey responses were submitted, 38 (34.5%) from OHSU and 72 (65.5%) from UNMC, with 109 (99.1%) complete enough to be included in analyses. The response rates for eligible participants at each institution were 38/124 (30.6%) at OHSU and 72/131 (55.0%) at UNMC. The Obs phase had 50 responses (45.9%), the WF phase had 21 responses (19.3%), and the JIT phase had 38 responses (34.9%). 

**Table 2:** Characteristics of IM Residents According to Study Phase

	**Total All Phases Combined** **(n=109)** ^**†**^ **n (%)**	**Baseline Observational Phase (** **n** **=50)** ^**†**^ **n (%)**	**Workflow Training Phase (** **n** **=21)** ^**†**^ **n (%)**	**Just-in-Time Training Phase(** **n** **=38)** ^**†**^ **n (%)**	**p value**
**Level of Training**					**0.029** ^**a**^
PGY-1	58 (53.2%)	28 (56.0%)	13 (59.1%)	17 (44.7%)	
PGY-2	27 (24.8%)	7 (14.0%)	4 (18.2%)	16 (42.1%)	
PGY-3	25 (22.9%)	15 (30.0%)	5 (22.7%)	5 (13.2%)	
**No POCUS Training during Medical School**	25 (22.9%)	10 (20.1%)	4 (19.0%)	11 (28.9%)	0.556^ b^
**No POCUS Training during Residency**	8 (7.3%)	7 (14.0%)	1 (4.8%)	0 (0.0%)	**0.039** ^b^
**Perception of POCUS Role in Patient Care**					0.400^**a**^
Very or Extremely Useful	76 (71.0%)	33 (67.4%)	13 (65.9%)	30 (79.0%)	
Moderately Useful	25 (23.4%)	12 (24.5%)	5 (25.0%)	8 (21.1%)	
Not at all or Slightly Useful	6 (5.6%)	4 (8.2%)	2 (10.0%)	0 (0)	

*PGY = Post Graduate Year

†n changes due to missingness

^a^Chi Square test

^b^Kruskal-Wallis test

**Table 3:** POCUS Use According to Phase of Training

**Study Variables **	Baseline Observational Phase (n=50)*	**Workflow Training** Phase (n=21)*	Just-in-Time Training Phase (n=38)*	**P Value **
**Point of Care Ultrasound (POCUS) Use **				
Percent with POCUS Cart or Handheld Access -n (% Yes)	46 (93.9%)	18 (85.0%)	38 (100.00%)	**0.002** **^b^**
Number (%) Co-Resident POCUS Use Not at all 1-3 times per month 1-3 times per week >3 times per week	15 (30.6%) 29 (59.2%) 3 (6.1%) 2 (4.1%)	8 (40.0%) 12 (60.0%) 0 (0) 0 (0)	6 (15.8%) 17 (44.7%) 12 (31.6%) 3 (7.9%)	**0.004** **^b^**
Number (%) Attending POCUS Use Not at all 1-3 times per month 1-3 times per week >3 times per week	31 (63.3% 12 (24.5%) 4 (8.2%) 2 (4.1%)	12 (60.0%) 5 (25.0%) 3 (15.0%) 0 (0)	15 (39.5%) 13 (34.2%) 7 (18.4%) 3 (7.9%)	0.340**^b^**
Personal/Team POCUS use During Ward Rotation – n (% Yes)	32 (65.3%)	13 (65.0%)	34 (89.5%)	**0.024** **^b^**
Number (%) of Patient Encounters You Used POCUS None 1-3 >4	5 (16.1%) 17 (54.8%) 9 (29.1%)	1 (7.7%) 11 (84.6%) 1 (7.7%)	4 (12.5%) 16 (50%) 12 (37.5%)	0.364**^b^**
Number (%) of Patient Encounters Your Team Used POCUS None 1-3 >4	1 (3.2%) 16 (51.6%) 14 (45.2%)	1 (7.7%) 8 (61.5%) 4 (30.8%)	4 (12.5%) 8 (25.0%) 20 (62.5%)	0.096**^b^**
**Documentation & Archiving Frequency **				
Mean % (SD) that reported they documented findings in a note	41.4 (39.9)	50.7 (46.8)	53.4 (33.9)	0.463^c^
Mean % (SD) that reported they saved images to an archiving system	17.2 (32.0)	40.7 (43.7)	57.0 (39.7)	**0.001** ^c^
Mean % (SD) that reported they did both	15.6 (30.9)	36.2 (45.2)	39.6 (35.5)	**0.039** ^c^
**Comfort Level with** **Skills** **^a^**				
Mean (SD) Comfort Performing POCUS	3.26 (1.08)	3.30 (0.92)	3.58 (0.89)	0.306^c^
Mean (SD) Comfort Interpreting POCUS	3.10 (1.06)	3.00 (1.03)	3.32 (0.93)	0.455^c^
Mean (SD) Comfort Interpreting POCUS into clinical decision making	3.26 (1.05)	3.50 (1.05)	3.45 (0.83)	0.543^c^
Mean (SD) Comfort Documenting POCUS into electronic medical record	2.88 (1.06)	2.20 (1.11)	2.82 (0.96)	**0.041** ^d^
Median (SD) Comfort Saving POCUS images to archiving system	2.56 (1.05)	2.40 (1.10)	2.95 (1.11)	0.124^ d^
**Summary Comfort Score**	15.06 (4.22)	14.40 (4.20)	16.11 (3.34)	0.247^d^

*n varies due to missingness^ ^^SD=Standard Deviation

^a^Scale: 1=Extremely uncomfortable; 2=Somewhat uncomfortable; 3=Neither comfortable nor uncomfortable; 4=Somewhat comfortable; 5=Extremely comfortable

^b^Chi Square c Kruskal-Wallis test

**Table 4:** Barriers and Facilitators of POCUS Use According to Study Phase

**Study Variables**	**Baseline** **Observational Phase** **(** **n** =50)* **Mean (SD)**	**Workflow Training Phase (** **n** =21)* **Mean (SD)**	**Just-in-Time Training Phase (** **n** **=38)** * **Mean (SD)**	**p value** **^†^**
**Barriers to POCUS Documentation** **^a^**				
Time due to competing responsibilities	3.21 (1.2)	3.08 (1.1)	3.26 (1.0)	0.885
Unclear institutional documentation standards	2.76 (1.1)	2.54 (1.3)	2.38 (0.9)	0.412
Unclear where to document POCUS in EHR	2.89 (1.1)	2.54 (1.3)	2.30 (1.2)	0.147
Unsure how to document/describe POCUS findings	2.66 (1.1)	3.08 (1.3)	2.40 (1.2)	0.210
Uncertain of POCUS findings	2.79 (0.9)	3.00 (1.2)	2.83 (1.0)	0.817
Concern for medical-legal liability	1.86 (1.0)	2.38 (1.5)	1.97 (1.1)	0.377
Someone else’s role on the team to document	1.41 (0.7)	1.92 (1.4)	1.5 (0.73)	0.217
**Barriers to POCUS Archiving** **^a^**				
Time due to competing responsibilities	3.33 (1.4)	3.00 (1.2)	3.03 (1.1)	0.579
Unclear institutional archival standards	2.85 (1.2)	2.54 (1.4)	2.25 (1.1)	0.186
Unclear where to archive POCUS images	2.89 (1.3)	2.62 (1.4)	2.29 (1.1)	0.190
Unsure how to archive POCUS images	3.37 (1.3)	2.69 (1.4)	2.64 (1.2)	0.086
Uncertain of POCUS findings	2.48 (1.1)	2.54 (1.1)	2.50 (0.9)	0.986
Concern for medical-legal liability	1.69 (0.9)	2.38 (1.4)	1.67 (0.9)	0.094
Someone else’s role on the team to archive	1.56 (0.8)	1.69 (1.1)	1.59 (0.8)	0.921
**Facilitators When Considering POCUS Use** **^b ^**				
Immediate access to handheld device	4.08 (0.9)	4.40 (0.8)	4.53 (0.7)	0.040
Immediate access to cart-based device	4.10 (0.7)	4.30 (0.7)	4.11 (0.8)	0.576
Team members confident with POCUS	4.37 (0.7)	4.50 (0.6)	4.08 (0.7)	0.065
Receipt of recent POCUS education	4.18 (0.8)	4.45 (0.6)	4.25 (0.7)	0.354
Access to POCUS resources	4.14 (0.7)	4.30 (0.8)	4.14 (0.8)	0.701

^a^Scale: 1=Insignificant barrier; 2=Small barrier; 3=Moderate barrier; 4=Significant barrier; 5=Very significant barrier

^b^Scale: 1=Strongly disagree; 2=Disagree; 3=Neither agree nor disagree; 4=Agree; 5=Strongly disagree

†Kruskal-Wallis test

Residents across the training spectrum were included, with the majority (53.2%, 58/109) being first year residents, as expected due to rotation structure. Twenty-five respondents (22.9%) reported they had not had POCUS training during medical school, and 7.3% (8/109) reported not having this training during residency (Table 2). In terms of perceived usefulness in practice, most residents (71.0%) thought POCUS was extremely or very useful, 23.4% reported it was moderately useful, and 5.6% reported it was slightly or not at all useful with no differences across study phases (Table 2).

Survey responses on POCUS use, documentation and archiving, and comfort are reported in Table 3. Respondents reported access to POCUS devices was high, ranging from 85% during the WF phase to 100% in the JIT phase (p=0.002). The percentage of respondents that reported personal or team POCUS use during the rotation increased from 65% in Phases 1 and 2 to 90% in the JIT phase (p=0.024), although the number of patient encounters using POCUS did not change for the individual (p=0.364) or team (p=0.096). The frequency of reported co-resident (residents from the same team) POCUS use increased across the phases (p=0.004), with approximately 40% of respondents reporting POCUS use at least weekly in the JIT phase. 

The percentage of respondents reporting they saved images to an archiving system improved across subsequent phases (17.2% to 40.7% to 57.5%, p=0.001). Frequency of self-reported documenting findings increased across study phases, but this was not statistically significant (mean=41.4% to 50.7% to 53.4%; p=0.463). Archiving images increased statistically from 17.2% to 40.7% and then to 57.0% (p=0.001) across study phases. Self-reported performance of both documentation and archiving increased from Obs phase to the WF and JIT phase (15% to 40%, p=0.039). POCUS-related comfort levels for performing, interpreting, integrating findings, documenting, and archiving were rated as moderate with no statistical differences in either individual variables or summary score according to study phases.

isplays barriers and facilitators related to POCUS use across study phases. Time was the greatest barrier for documentation and archiving due to competing responsibilities, which was considered a moderate barrier and not affected by study phase. The least consequential barrier for documentation and archiving was it being Someone Else’s Role on the Team, which was an insignificant/small barrier not affected by study phase. Respondents rated all facilitators of POCUS use high (>4.1 on a five-point scale). Only Immediate Access to POCUS Devices changed across study phases (4.08 to 4.40 to 4.53, p=0.040).

Three factors correlated with personal POCUS use and two factors correlated with team POCUS use (Table 5). Personal POCUS use had a moderate correlation with team POCUS use­ (r_s_=0.431; p<0.001). The Summary Comfort (score r_s_=0.293, p=0.01), and co-resident POCUS use (r_s_=0.242; p=0.035) were weakly correlated with personal POCUS use. Attending POCUS use , availability of type of POCUS devices (handheld vs. cart-based) and perceptions of POCUS usefulness in patient care was not correlated with personal POCUS use. Team POCUS use had a strong correlation with attending POCUS use (r_s_=0.523; p<0.001) and a moderate correlation with co-resident POCUS use (r_s_=0.496; p<0.001) and personal POCUS use (r_s_=0.431, p<0.001). We found no correlation between frequency of documenting and archiving images and comfort and skill with POCUS variables (all p values >0.379).

**Table 5:** Factors Correlated with Personal POCUS Use All Study Phases Combined

**Study Variables**	**Correlations with Personal POCUS Use**	p value*	**Correlations with Team POCUS Use**	p value*
Personal POCUS Use	**--**	**--**	0.431	**<0.001**
Team POCUS Use	0.431	**<0.001**	**--**	**--**
Co-Resident POCUS Use	0.242	**0.035**	0.496	**<0.001**
Attending POCUS Use	0.154	0.186	0.523	**<0.001**
Availability of POCUS Device†	-0.005	0.964	-0.024	0.835
Perception of POCUS Usefulness in Patient Care	0.181	0.118	0.028	0.810
Summary Comfort Score	0.293	**0.010**	-0.157	0.177

*Spearman’s Correlation Coefficient

†POCUS devices were essentially universally available

## Discussion

Spaced repetition is vital to buffering against psychomotor skill loss, yet its effect on POCUS skill acquisition remains unknown. Our study is one of the first attempts to assess the effects of a JIT POCUS training built into existing resident curriculum at multiple sites, and explore resident perception of POCUS documentation. We found that both self-reported personal and team use of POCUS increased significantly between phases where active learning was greatest. We additionally noted that co-learning seems to play an important role in the performance of POCUS. Personal POCUS use correlated with team and co-resident POCUS use, suggesting that role-modeling and peer-coaching may encourage behavioral changes among team members. Furthermore, the strongest correlation we found was between team POCUS use and attending POCUS use, which suggests that faculty tend to engage learners as a team, rather than individually. While this is encouraging that faculty can provide supportive scaffolding and foster POCUS use, POCUS-facile faculty remain in short supply and are a primary limiting factor to residents obtaining more training 12.

Both UNMC and OHSU offered a POCUS workshop for categorical first year residents but residents’ subsequent exposure to POCUS was variable, resulting in only moderate confidence in their POCUS skills. While there were modest changes in confidence with POCUS following a JIT training, we noted that a single training was subject to the same skill loss observed in workshop experiences. Thus, we theorize that more frequent training experiences weaved into the resident curriculum combined with improved supervision (faculty and senior resident level) will likely aid in the retention of information and skills as posited by Spaced Repetition Theory. Therefore, it is important for curriculum designers to simultaneously focus on a “bottom-up” approach (junior medical trainees, including medical student and junior residents) with a “top-down” approach to target potential trainers (for example, senior residents and faculty). Institutional investment in faculty development will be vital for the growth of POCUS within internal medicine and other disciplines in the early stages of adopting POCUS. For locales with limited POCUS-trained faculty, the use of peer or near-peer training has proven successful 25, 26, 27. 

With increasing POCUS use, developing systems to archive POCUS images and document findings can allow for asynchronous supervision of residents using POCUS in clinical care. The “Workflow” phase of our program attempted to improve resident documenting habits by offering a standardized independent process to the residents. We found that documenting POCUS findings and archiving images increased significantly between study phases, which suggests that the JIT training had an impact on POCUS use among those in the study population. However, “time” was cited as the biggest barrier to documentation activities and was unaffected by study phase. Again, we hypothesize that faculty “expectation” and co-learning may be helpful to improve documentation habits and change a culture where absence of documentation remains the norm.

The strengths of this study include having two IM residency training sites with all three years of IM training, which improves our findings’ generalizability. We captured data from different cohorts of residents, including categorical and non-categorical trainees, during a 10-month period. 

Despite these strengths, the study had several limitations that deserve mention. First, the survey required the residents to think back over the course of their rotation, and thus could be affected by recall bias, which may affect the accuracy of results 28. Second, the study relied on self-reported behaviors, which may not reflect their actual clinical behaviors, also affecting accuracy. Future studies could mitigate these limitations by evaluating more objective outcomes, such as the number of exams archived, frequency of interpretation report completion, and quality of saved images before and after educational interventions. This may require better streamlining of image management workflow. For example, since the completion of this study, the authors have established automatic exporting of saved POCUS exams for archiving and better “policing” of trainees to ensure interpretation reports are completed. Finally, the two sites used similar, but slightly different implementation strategies for the WF and JIT interventions, which may have impaired the internal consistency of the study. This was done for pragmatic reasons due to the different rotation structures and ultrasound and EMR systems. Importantly, we found no significant differences when comparing survey responses between the two sites, so the impact was likely negligible. Such differences reflect the real-world, site-specific challenges that institutions will likely face when implementing POCUS interventions. 

## Conclusion

In conclusion, following implementation of POCUS workflow and just-in-time skills training interventions we found moderate, but statistically significant, improvements in residents’ self-reported documentation, image archiving, and team POCUS use. Attending POCUS use was strongly correlated with that of the resident team, so more supervising faculty need POCUS training to provide scaffolded learning opportunities and role-modeling for trainees to build upon foundational skills. Future research should focus on how best to ensure archiving images and documenting examinations occurs in a training environment where most IM supervising physicians are not proficient in either the performance or interpretation of POCUS. 

## Disclosure Statement 

The authors declare that they have no competing interests.
